# A Generic Mechanism for Adaptive Growth Rate Regulation 

**DOI:** 10.1371/journal.pcbi.0040003

**Published:** 2008-01-11

**Authors:** Chikara Furusawa, Kunihiko Kaneko

**Affiliations:** 1 Department of Bioinformatics Engineering, Graduate School of Information Science and Technology, Osaka University, Suita, Osaka, Japan; 2 Complex Systems Biology Project, ERATO, JST, Komaba, Meguro-ku, Tokyo, Japan; 3 Department of Pure and Applied Sciences, University of Tokyo, Komaba, Meguro-ku, Tokyo, Japan; University of California Los Angeles, United States of America

## Abstract

How can a microorganism adapt to a variety of environmental conditions despite the existence of a limited number of signal transduction mechanisms? We show that for any growing cells whose gene expression fluctuate stochastically, the adaptive cellular state is inevitably selected by noise, even without a specific signal transduction network for it. In general, changes in protein concentration in a cell are given by its synthesis minus dilution and degradation, both of which are proportional to the rate of cell growth. In an adaptive state with a higher growth speed, both terms are large and balanced. Under the presence of noise in gene expression, the adaptive state is less affected by stochasticity since both the synthesis and dilution terms are large, while for a nonadaptive state both the terms are smaller so that cells are easily kicked out of the original state by noise. Hence, escape time from a cellular state and the cellular growth rate are negatively correlated. This leads to a selection of adaptive states with higher growth rates, and model simulations confirm this selection to take place in general. The results suggest a general form of adaptation that has never been brought to light—a process that requires no specific mechanisms for sensory adaptation. The present scheme may help explain a wide range of cellular adaptive responses including the metabolic flux optimization for maximal cell growth.

## Author Summary

Adaptation of living systems to various environmental conditions is one of the most universal phenomena in biology. As is well known from the paradigmatic case in the Escherichia coli lac-operon system, cellular adaptation is generally understood as a physiological shift that is elicited by regulation of genes with specific signal transduction machinery. However, here is an unsolved paradox. If such strategy is the only means by which cells can adapt to a different environment, cells cannot survive a novel environment before a signal transduction apparatus has a chance to evolve. Some form of nonspecific adaptation must allow cells to grow robustly in the novel environment, as is also suggested by recent experiments. This is natural considering that a huge set of signal transduction mechanisms would otherwise be needed for all potential environmental conditions that cells may face. Our theoretical study demonstrates that, in fact, changes in gene expression pattern can be adaptive; i.e., a state most favorable for cells' survival is selected without explicit hardwired regulatory circuits. This occurs inevitably for any cells that grow under stochastic gene expression. Our mechanism is generic and explains why cells adapt and grow optimally in a variety of environments, taking advantage of stochasticity.

## Introduction

Cells adapt to a variety of environmental conditions by changing the pattern of gene expression and metabolic flux distribution. These adaptive responses are generally explained by signal transduction mechanisms, where extracellular events are translated into intracellular events through regulatory molecules. For example, the Lac operon of Escherichia coli encodes proteins involved in lactose metabolism, and expression of the operon is controlled by a regulatory protein so that, when lactose is available, these proteins are expressed in an efficient and coordinated manner [1]. In general, adaptive responses are depicted by a prewired logic circuit that takes an environmental condition as an input and gene expression as an output.

However, such program-like descriptions may not always apply, since the number of possible environmental conditions to which a cell must adapt is so large compared to the limited repertoire of gene regulatory mechanisms. For example, experiments using phenotype microarrays [2] revealed that E. coli cells grow in hundreds of environmental conditions, including different carbon and nitrogen sources and stress environments, in which they are distinctly altered states of gene expression [3]. Considering that the number of E. coli genes categorized as “signal transduction mechanisms” in the genome is less than a few hundred [4], it is less plausible that cells have gene regulatory programs to adapt to such a variety of environmental conditions. Indeed, in case of bacterial growth, a general adaptation process that occurs over generations seems to exist in addition to adaptation through gene regulation by signal transduction mechanisms [5,6].

Two recent studies indicated the possibility that cells can respond to environmental changes adaptively without pre-programmed signal transduction mechanisms. Braun and colleagues demonstrated using yeast cells that even when the promoter of the essential gene (HIS3) is detached from the original regulatory system, expression of the gene is regulated adaptively in response to environmental demands [7,8]. Furthermore, Kashiwagi et al. demonstrated that E. coli cells select an appropriate intracellular state according to environmental conditions without the help of signal transduction mechanisms [9]. There, an artificial gene network composed of two mutually inhibitory operons was introduced into E. coli cells, so that states of gene expression are bistable. These authors found that the cells shift to the adaptive cellular state by expressing the gene required to survive in the environment. They also demonstrated that the selection of the adaptive attractor between bistable states by noise is possible by introducing phenomenological activity that governs the synthesis and degradation of protein.

In the present study, we move beyond the two-gene model and demonstrate that cells select states most favorable for their survival among a large number of other possible states as an inevitable outcome of the very fact that cells grow and that gene expression is inherently stochastic [10–14]. By studying a model that consists of a protein regulatory network and a metabolic reaction network, we show that cellular states with high growth rates are selected among a huge number of possible cellular states, and this selection is only mediated by fluctuations of gene expressions. This selection of a higher growth state is theoretically explained by noting that a state with lower growth speed is more influenced by stochasticity in gene expression, so that it is easily kicked away, triggering a switch to a state with a higher growth rate. We show that there is generally a negative correlation between the rate of noise-driven escape from a given state and the cellular growth rate. Due to this negative correlation, an optimal growth state is selected spontaneously. Noting the generality of this selection mechanism, we provide a possible answer to the question how cells generally adapt to a larger variety of environmental conditions by changing their gene expression pattern, even without a specific signal transduction mechanism.

## Model

A schematic representation of our model is shown in Figure 1. It consists of two networks, i.e., a regulatory network that controls expression levels of proteins through each other, and a metabolic reaction network whose fluxes are regulated by the expression levels of the proteins. The internal state of a cell is represented by a set of expression levels of *n* proteins (*x*_1_,*x*_2_, ··· , *x*_n_) and concentrations of *m* metabolic substrates (*y*_1_,*y*_2_, ··· , *y*_m_). The change in expression levels of proteins over time is determined by (i) the synthesis of proteins, (ii) dilution of proteins by the cell volume growth, and (iii) molecular fluctuation arising from stochasticity in chemical reactions. The dilution of proteins is proportional to the growth rate of cell volume *v_g_*, which is determined by the metabolic fluxes. Also, we assume that the rates of protein synthesis are proportional to the growth rate *v_g_*. This assumption is natural and is necessary to maintain a steady state, since the decrease in protein concentration by dilution due to the cell growth has to be compensated by synthesis (the biological plausibility of the assumption will be discussed in the section Discussion). Thus, we write the dynamics of expression level of the *i*-th protein as follows:

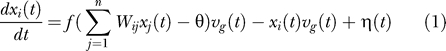



**Figure 1:**
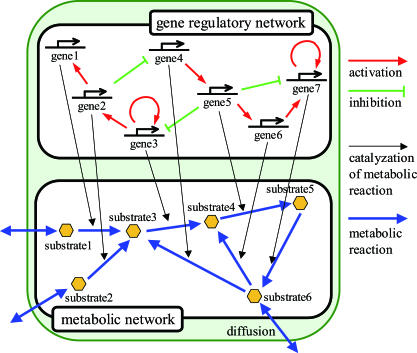
Schematic Representation of Our Cell Model The model consists of two networks, i.e., a gene regulatory network and a metabolic network. As a schematic example, simple networks consisted of *n* = 7 genes and *m* = 6 metabolic substrates are shown. The red arrows in the regulatory network represent activation of expressions, while green lines with blunt ends represent inhibition. The arrows from a gene to itself mean autoregulation of expressions. As a result of these regulatory interactions, the dynamics of expression levels of proteins have multiple attractors. The metabolic reactions, represented by blue arrows, are controlled by expression levels of corresponding proteins. The correspondence between metabolic reactions and gene products (proteins) are shown by the thin black arrows. The regulatory matrix *W_ij_* of the presented network takes *W*_21_ = *W*_32_ = *W*_33_ = *W*_45_ = *W*_56_ = *W*_67_ = *W*_77_ = 1, *W*_24_ = *W*_53_ = *W*_57_ = −1, and 0 otherwise. The reaction matrix *Con*(*i*,*j*,*k*) metabolic network takes a value 1 for the elements (1,3,1)(2,3,2)(3,4,3)(6,3,4)(4,5,5)(6,4,6)(5,6,7), and 0 otherwise. The choice of *n* = 7 in the figure is only for schematic illustration, and in the actual simulation we used much larger networks with *n* = 20 ∼ 100. In the present paper, we adopt a much larger network with *n* = 96 genes and *m* = 32 substrates.

The first and second terms in r.h.s. represent synthesis, dilution of the protein *i*, respectively. In the first term, the regulation of protein expression levels by other proteins are indicated by regulatory matrix *W_ij_*, which takes 1, 0, or −1, representing activation, no regulatory interaction, and inhibition of the *i*-th protein expression by the *j*-th protein, respectively. The synthesis of proteins is given by the sigmoidal regulation function *f*(*z*) = 1 / (1 + *exp*(−*μz*)), where *z* = (∑*W_ij_x_j_*(*t*) − *θ*) is the total regulatory input with the threshold *θ* for activation of synthesis, and *μ* indicates gain parameter of the sigmoid function. The regulatory interactions are determined randomly with the rate *ρ_a_*, *ρ_i_*, indicating the connection rate of excitatory paths and inhibitory paths, respectively.

The last term of r.h.s. in Equation 1 represents the molecular fluctuation. For a specific form of the noise, we assume that there are fluctuations on the order of 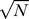 for reactions involving *N* molecules, then we add a noise term 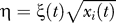 , where *ξ*(*t*) denotes Gaussian white noise with the amplitude *σ*. In this model, we assume that the amplitude of the noise is independent of the synthesis and dilution terms of proteins, since the inclusion of noise source that depends on the rates of synthesis or dilution does not change the simulation results qualitatively.

Temporal changes in concentrations of metabolic substrates are given by metabolic reactions and transportation of substrates from the outside of the cell. Each metabolic reaction is catalyzed by a corresponding protein. Some nutrient substrates are supplied from the environment by diffusion through the cell membrane, to ensure the growth of a cell. Here, the dynamics of *i*-th substrate concentration *y_i_* is represented as:

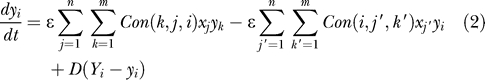
where *ɛ* indicates the coefficient for the metabolic reactions, and *Con*(*i*,*j*,*k*) represents the reaction matrix of the metabolic network, which takes 1 if there is a metabolic reaction from *i*-th substrate to *k*-th substrate catalyzed by *j*-th protein, and 0 otherwise. The first and second terms of r.h.s. correspond to synthesis and consumption of *i*-th substrate by metabolic reactions, respectively. The third term of r.h.s. represents the transportation of the substrate through the cell membrane, which is approximated by the linear term in the diffusion process with a diffusion coefficient *D*. *Y_i_* is a constant representing the concentration of *i*-th substrate in the environment. The concentration *Y_i_* is nonzero only for nutrient substrates.

The cellular growth rate *v_g_* is determined by the dynamics in the metabolic reactions. We assume that some of metabolic substrates are necessary for cellular growth, and the growth rate *v_g_* is determined as a function of the concentrations of them. Several choices of the function are possible, and the results to be discussed are generally observed as long as the growth rate varies drastically depending on the concentrations. Here we assume that the growth rate is proportional to the minimal concentration among these necessary substrates. In other words, among *m* metabolic substrates there are *r* substrates (*y*_1_,*y*_2_, ··· , *y_r_*) required for cellular growth, and the growth rate is represented as *v_g_* ∝ min(*y*_1_,*y*_2_, ··· , *y_r_*)

The basic requirements of our model are summarized in the first column of Table 1. In our model, we use specific forms to realize these requirements, as summarized in the second column of Table 1 and Equations 1 and 2, while some generalizations are possible as long as they do not harm the requirements, as given in the third column of the Table 1.

**Table 1:**
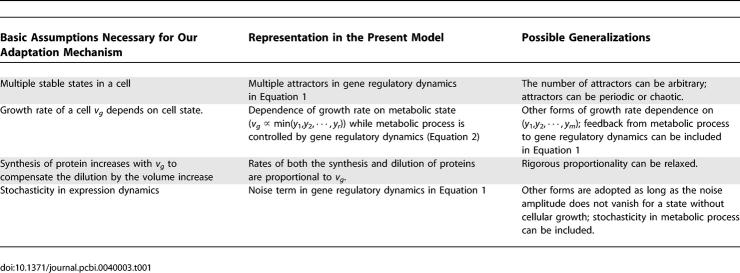
Summary of Basic Requirements for Our Adaptation Mechanism

The requirement for the network to exhibit multiple attractors entailed some constraints on the range of the parameters of the network topology. The parameter values of network connectivity (e.g., *ρ_a_* ∼ .03 and *ρ_i_* ∼ .03) are thus chosen, which may correspond to Kauffman's ordered regime [15]. Also, some positive auto-regulation paths (*W_ii_* = 1) are included, which facilitate coexistence of multiple attractors (in fact, there is a sufficient number of auto-regulation paths even for E. coli [16]).

We carried out numerical experiments with the model using several sets of parameter values obeying the above constraints that allows for multiple attractors, and evaluated thousand of different randomly generated reaction networks. We found that the adaptation processes triggered by noise are generally observed, as long as the requirements in Table 1 are satisfied. In the next section, we present the typical behaviors obtained by using networks consisting of *n* = 96 proteins and *m* = 32 metabolic substrates.

## Results

In Figure 2, an example of such a selection process of states is shown by taking the noise amplitude *σ* = 0.2. Time series of expression levels of arbitrarily chosen proteins and growth rate of the cell are plotted in Figure 2A and 2B, respectively. In the example, cells are initially put at a state with a low growth rate. In such a state, stochasticity dominates the time evolution of protein levels.

**Figure 2:**
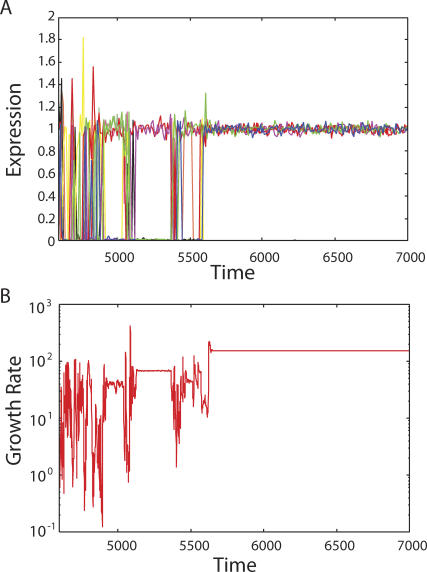
Selection of a Higher Growth State by Noise (A) Time series of protein expressions *x_i_*(*t*). Ten out of 96 protein species are displayed. The vertical axis represents the expression levels of proteins, and the horizontal axis represents time. (B) Change in growth rate *v_g_* observed during the time interval shown in (A). Initially, the growth rate of the cell fluctuates due to the highly stochastic time course of protein expression. After a few short-lived nearly optimal states (c.f. 4,800 ∼ 5,600 time steps), the cell finds a state of protein expression that realizes a high rate of growth. The parameters are *θ* = 0.5, *μ* = 10, *ρ_a_* = *ρ_i_* = 0.03, *ɛ* = 0.1, and *D* = 0.1. In addition, we enhanced the rate of positive autoregulatory paths, i.e., *W_ii_* = 1 for *i*-th gene, so that the regulatory network has multiple attractors. In the simulations, 30% of activating paths are chosen as autoregulatory paths.

After itinerating among various expression patterns, the cellular dynamics finds itself in a state with a higher growth rate. Such transition repeats until the growth rate becomes sufficiently high. Once a gene expression pattern supporting the optimal growth is reached, the system maintained it over time.

This selection of higher growth states is observed for all the thousand networks we simulated. It also works independently of initial conditions. As the final state depends on the initial condition, we have computed the distribution of the final growth rate reached from randomly chosen initial conditions. The distribution of final growth rate thus obtained is plotted in Figure 3A. In the case without noise, i.e., the noise amplitude *σ* = 0, the cellular state rapidly converges deterministically into an attractor. In such a case, the final growth rates exhibit a broad distribution as shown in Figure 3A, representing a wide variety of the final cellular states. In contrast, under presence of noise (*σ* = 0.2), the final growth rates exhibit a relatively sharp distribution, due to the selection process of faster growth states, as we have seen in Figure 2.

**Figure 3:**
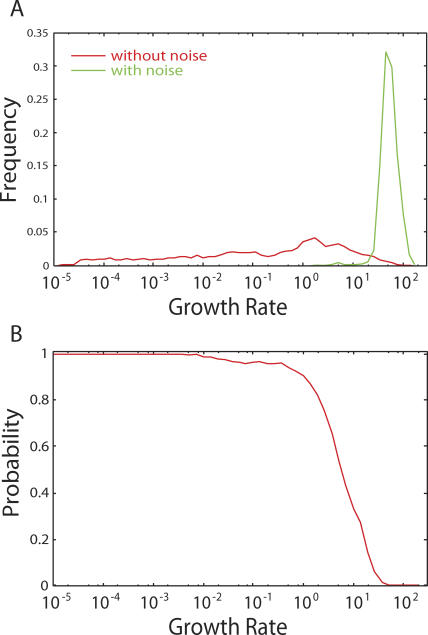
Distribution of Growth Rate and Escape Probability from an Attractor (A) The distribution of growth rate. Starting from randomly chosen 10^5^ initial conditions, the distribution of growth rates after 10^5^ time steps are computed with and without noise (*σ* = 0.2). (B) Relationship between the growth rate *v_g_* and the probability to escape an attractor within a certain period of time. The probability is computed by 10^5^ trials starting from randomly chosen initial conditions. After a cell reaches a stable state, noise (*σ* = 0.2) is added, and the time it takes the cell to escape from the corresponding attractor is measured. The *y-*axis represents the probability that the cellular state is kicked out of the original state within 10^3^ time steps, and the horizontal axis shows the growth rate *v_g_* of the original state.

Note that once one of the expression patterns is selected as an attractor, the flux pattern on the metabolic network is uniquely determined. As a result, the cellular growth rate *v_g_* is also fixed, which in turn affects the protein expression dynamics. Here the influence of noise depends on the growth rate *v_g_* for each attractor. When *v_g_* is small, the deterministic part of protein expression dynamics (i.e., the first and second terms of r.h.s. in Equation 1) is small, so that the stochastic part in the dynamics is relatively dominant in the protein expression dynamics. Then, the probability to escape the attractor due to fluctuation is large. In contrast, when the growth rate *v_g_* is large in the attractor, the magnitude of the deterministic part of expression dynamics is larger than that of the stochastic part. As a result, the probability to escape the state becomes small (even if the attractor of regulatory dynamics is not a fixed point but oscillatory, our argument follows by considering the minimal, or average, growth rate of each oscillatory state). It should be noted that all the protein concentrations are not necessarily higher for an adaptive state. Some proteins will increase their concentrations, while others will not. For an adaptive attractor, the overall synthesis rate of proteins is increased, but the overall concentration is not necessarily increased since the dilution of proteins by cell growth is also increased.

In Figure 3B, the relationship between the growth rate *v_g_* and the probability of an escape to an attractor within a period of time is displayed. The probability is higher as the growth speed of cell is lower. It follows naturally from this relationship that cells drift with a directional bias toward a higher growth rate. Hence, as long as the deterministic part of gene expression (i.e., synthesis minus dilution) increases with the growth rate *v_g_* while the noise amplitude has a *v_g_*-independent part, the selection of attractors with higher growth rates generally follows.

The emergence of the selection process as presented in Figure 2 is not restricted to a specific environmental condition. Instead, the mechanism is a general one ensured by the physical limitation of the replicating system. The mechanism makes it inevitable for the cells to seek states with (nearly) optimal growth independently of environmental context. To show the adaptation process over several environmental conditions, we have computed the temporal evolution of our model, under changing nutrient conditions. This was achieved by updating the concentrations *Y_i_* of a set of substrates that had nonzero *Y_i_* in a few perturbations at successive time points. We have plotted in Figure 4 a time series of protein expressions and the growth rate, while environmental conditions are changed at the time points indicated by arrows. After the environmental changes, the fluctuation in expression dynamics is observed. This increase in fluctuation continues, until the cell finally finds a state that ensures a high growth rate. Adaptation to a novel environment is thus possible.

**Figure 4:**
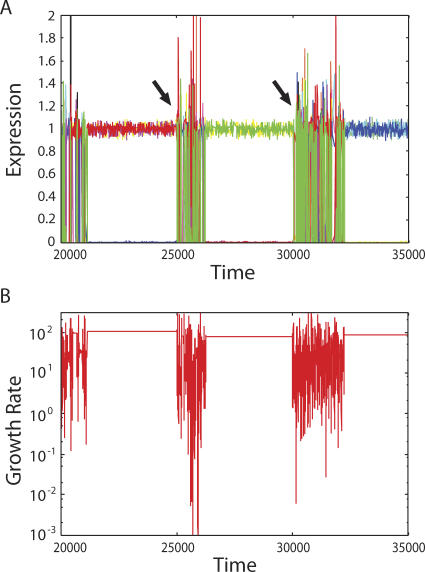
Adaptation Process over Several Environmental Conditions (A) Time series of protein expressions *x_i_*(*t*) when the environmental condition is altered. The environmental conditions, i.e., substrates having nonzero *Y_i_*, are changed at time points indicated by arrows. (B) Change of growth rate *v_g_* in the same time interval as (A). After the environmental changes, both expression levels of all proteins and the growth rate start to fluctuate until the cell finds a state of protein expression that realizes a high growth rate. In the simulation, the noise amplitude *σ* = 0.2.

Next, we investigate how this noise-driven adaptation depends on the noise amplitude. In Figure 5, the final growth rate *v_g_* is plotted against the noise amplitude *σ*. For small noise amplitude (*σ* < 10^−2^), the final growth rates are broadly distributed, since cells cannot escape from the first attracting state that they encounter. On the other hand, when the noise amplitude is larger (*σ* > 1), the final growth rates again exhibit a broad range distribution, because the cellular state continues to change without settling into any attractor. In the intermediate range of the noise strength 10^−2^ < *σ* < 1, those cellular states are selected that are associated with significantly higher growth rates than those found in the other noise ranges. This shift of the final growth rate is due to the selection of cellular states by fluctuations, as shown in Figure 2.

**Figure 5:**
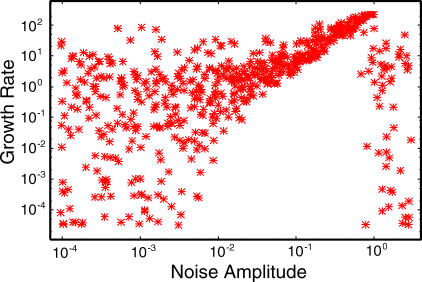
The Relationship between the Noise Amplitude *σ* and the Growth Rate *v_g_* Starting from randomly chosen initial conditions against the noise amplitude *σ* ranging 10^−4^ < *σ <*3, the growth rates *v_g_* after 10^5^ time steps are plotted. In the intermediate range *σ* of the noise strength 10^−2^ < *σ <*1, cellular states with high growth rates are selected among a huge number of possible cellular states, as depicted in Figure 2.

Stability of a given attractor against noise is estimated by whether the first two terms in Equation 1 are larger than the noise term. One can roughly estimate that the stability changes at around *v_g_* × *O*(*x*) ∼ *σ* ^2^, where *x* represents the protein expression represented in Equation 1. If the former term is larger for attractors with higher growth rates, and smaller for other attractors with lower growth rates, then the former attractors will be selected. Considering that the term *O*(*x*) is about 0.1 ∼ 1, higher growth rates are selected when *σ* ^2^ exceeds min(*v_g_*)(0.1 ∼ 1), while the selection no longer occurs when *σ* ^2^ > max(*v_g_*)(0.1 ∼ 1) where all the states are visited randomly (here max and min represent the maximum and minimum of *v_g_* over attractors, respectively). The selection works within the range of noise amplitude min(*v_g_*) < *σ* ^2^ / (0.1 ∼1) max(*v_g_*). This estimate is consistent with the numerical simulation.

## Discussion

Numerical simulation and analysis of our model demonstrate how stochasticity in cellular reaction dynamics results in the selection of a stable gene expression pattern that supports higher growth rates. The selection works for any initial cellular states and environmental conditions. The results presented in this paper generally appear as long as the conditions in Table 1 are satisfied, i.e., coexistence of multiple attractors, dependence of growth rate on attractors, increase of cellular reaction process with the growth speed, and stochasticity in reaction dynamics.

As long as these requirements are satisfied, our results on adaptation are obtained, independently of the details of the model, such as parameters and model equations. To be specific, we have confirmed the robustness of our result against the following changes in the model.

**Parameter values of reaction dynamics.** The results presented in this paper are robust with respect to parameter changes in reaction dynamics, as long as the basic requirements in Table 1 are satisfied. For example, if the reaction coefficient of metabolic reactions changes from *ɛ* = 0.1 to 10, the selection of higher growth states still occurs, although the time necessary to approach the final high-growth states may depend on the parameter values.

**Determination of growth rate by metabolites**. In the present model, we assume that some metabolites are required for cellular growth and a metabolite having minimum concentration among these metabolites limits the growth rate. Thus, we use the simple rule that the growth rate *v_g_* is determined to be proportional to the minimum concentration of these metabolites. However, such specific form on how the growth rate depends on metabolites is not important for our results; instead, the same results are obtained as long as the growth rate is somehow determined by metabolite concentrations.

**Network properties**. We confirmed robustness of our result against the change in the properties of regulatory and metabolic networks, such as the path density or distribution of number of paths (including scale-free distribution). The adaptation by noise generally appears when there are multiple attractors in the regulatory dynamics.

**Stochasticity in metabolic reactions**. In our model, the fluctuation of metabolites concentrations is ignored, considering that the numbers of metabolite molecules are sufficiently large. However, inclusion of fluctuations of metabolite concentrations does not alter the adaptation process presented here.

**Regulation of protein expression by metabolite**. Some metabolites are known to regulate the protein expression dynamics, such as lactose and galactose, to transmit information on environmental conditions to regulatory dynamics. However, we do not incorporate such feedback regulations from metabolites into the model, since the essence of our results is to demonstrate that the adaptation process to any environmental condition is possible by the stochastic nature of regulatory dynamics even without such feedback regulations. It can be expected that the inclusion of such feedback from metabolite will not alter the adaptation process we proposed, as long as the requirements in Table 1 are satisfied. Similarly, inclusion of different types of proteins, such as regulatory factors, catalysts of metabolic reactions, and building blocks does not harm the adaptation process.

The selection of an attractor with higher growth rates works when the cellular states are switched by stochasticity in protein expressions and there is a negative correlation between the growth speed and residence time of each cellular state. In our model, the negative correlation is incorporated through the property that both the synthesis and dilution of proteins are proportional to the growth rate, while the noise amplitude is independent of it.

The rigorous proportionality of protein synthesis and dilution rate to the growth rate can be relaxed. Indeed, the present adaptation mechanism works as long as there is a positive correlation between the synthesis rate and the cell volume growth rate. There is some experimental support. For example, the positive correlation between the rate of protein synthesis and the growth rate was demonstrated in some microorganisms [17,18]. Furthermore, the fact that the intracellular protein concentrations are relatively unchanged in cells against the change in the cell growth rate [17,19] indicates that the synthesis and dilution of proteins in a cell are balanced. Since the dilution of proteins is proportional to the growth rate, this also supports the proportionality between the protein synthesis and the cell growth rate. Of course, one could include the degradation process of proteins in addition to the dilution. Even though the growth-rate dependence of the degradation process is not clear, the present adaptation mechanism still will work as long as the growth-dependent dilution dominates the decrease of protein concentrations.

Even if the noise form in gene expression is varied, the attractor selection will work as long as the noise amplitude does not vanish with the growth rate, or, in other words, as long as a certain amplitude of the noise is maintained in the non-adaptive state. For example, we have simulated a model with another noise term, 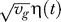 , in addition to the noise in Equation 1, and confirmed that the present adaptive attractor selection still works.

On the other hand, if the variance of total noise decreased linearly with the growth rate *v_g_* and vanished at *v_g_* = 0, the present selection would not work. However, as long as there is a basal process for the protein synthesis (and degradation) even when a cell does not grow, there should exist a growth-independent part in the noise as in Equation 1. Although that part of noise has not been measured separately, the fact that the synthesis of mRNAs, proteins, and metabolites are maintained even in the stationary phase of a cell [20] suggests that there exists a growth-independent part in the noise term.

As for the description of stochastic dynamics in cells, there are two major methods, i.e., continuous dynamics (Langevin description) as adopted here and discrete particle dynamics. An efficient algorithm for the latter description is the Gillespie method [21]. To confirm the validity of our result, we have also simulated a stochastic model using the Gillespie method. Due to technical limitations in the computational speed, we have simulated a simpler model with a few degrees of freedom that allows only for two attractors in the regulatory dynamics. We observed that the attractor with a higher growth rate is selected, in agreement with the simulation of the Langevin equation (Equation 1), as long as the noise does not vanish with the rates of synthesis and degradation of proteins. This suggests that the present attractor selection works if the number of molecules in a cell is not so large.

The magnitude of protein expression noise quantified by coefficient of variation could be on the order of 0.1 ∼ 0.01, as shown in [22]. In some cases it is suggested that the fluctuation is large enough to force cells back and forth between discrete states [23]. This magnitude of noise is within the estimated range required for the attractor selection, although separate measurement of the basal noise is necessary to complete the estimate.

The present study provides a possible explanation for the establishment of the optimal growth rate in the metabolic reaction networks, proposed by Palsson and his colleagues [24–26]. Their study suggested that a metabolic network is organized so that the growth rate is optimized under given conditions. For example, it was shown that E. coli strains with a deletion of a single metabolic gene can adapt to several environmental conditions, and that the value of the final growth rate is consistent with that calculated as an optimal growth rate in these perturbed metabolic networks and environmental conditions [26]. The observed adjustments of metabolic fluxes often occur within several days, suggesting that such an adaptation process is not caused by selection of mutants having a higher growth rate under the given condition. These bacteria adjust their intracellular state to optimize the growth rate, even though they have never experienced such an environment.

In fact, the attractor selection presented in this paper provides a mechanism for selecting a cellular state with an optimal growth rate, over a variety of environmental conditions. An important point here is that the presented mechanism requires no fine-tuning of regulatory mechanisms. As long as the cellular states are perturbed sufficiently by the stochasticity in gene expressions, a negative correlation between the growth rate and the escape probability from the corresponding cellular state is established. Thus, we propose that adaptive attractor selection may be at work behind the observed regulations of metabolic fluxes leading to optimal growth rate.

The merit of the present adaptive attractor selection induced by, and optimizing, growth lies in its generality. The mechanism can work without fine-tuning through evolution. Indeed, it makes adaptation possible to a novel environment that the species has not experienced in the course of evolution. This is important given that organisms have to survive by adapting to new environments even before a specific signal transduction network has been developed. Our mechanism provides such general and nonspecific “proto-adaptation.”

Of course, there are demerits in our mechanism also. If the difference in the growth rates between the two adaptive states is small, the present mechanism cannot distinguish them. Either of these states can be selected. Hence it does not work for very “fine-grained” selection between attractors conferring closely similar growth rates. Also, the selection process proposed here is not very efficient. The time required for the adaptation can be long. For example, in the case shown in Figure 2, a large number of generations is needed to reach the adapted state with the optimal growth rate. This long adaptation time is a disadvantage of the present noise-driven attractor selection, compared with the fine-tuned signal transduction mechanisms. However, such long adaptation time for novel environments may not be fatal for organisms in nature, since not every cell has to adapt to such environments. For example, let us consider the case that a population of a large number of cells encounters a novel environment. Even if the average time required for noise-driven adaptation is long, some cells in the population will be able to find an adapted state within a single or a few generations, given the stochastic nature of the present mechanism. Then these cells start to increase their population. Adaptive response at the population level progresses rather fast, even when each adaptation at the single cell level is inefficient.

The reason that the present adaptation process takes so long a time is that we have considered selection process over 1,000 attractors, to demonstrate clearly how it works. On the other hand, if the number of attractors is few for each given environment, the selection process is generally completed within a generation or a few. Indeed, the selection over a few attractors may be sufficient to explain adaptation to most novel environments.

Also, the time required for adaptation depends on the choice of network. Here we used regulatory networks generated randomly. By using an organized network, the attractor landscape, such as ruggedness, will be changed, so that the adaptation time can be radically reduced. For example, it is interesting to study the evolution of attractor landscapes under the present noise-driven adaptation. We expect that some nontrivial characteristics in attractor landscapes (e.g., funnel structures) would emerge to enhance fast and accurate response to environmental changes, which may help us to understand how existing signal transduction mechanisms have evolved. The relationship between adaptation dynamics and the characteristics of attractor landscapes is an important future topic.

In the adaptation process proposed here, existence of multiple attractors is necessary. However, there is little evidence that the regulatory network within a cell has multiple attractors, so far [23,27–29]. With regard to the gene regulatory network, there is a certain amount of (positive) feedback regulatory interactions [16], which can result in multi-stability of regulatory dynamics. On the other hand, experimental confirmation of the existence of multiple attractors in regulatory dynamics is still difficult, since simultaneous and single-cell level measurements of multiple genes/proteins are necessary for such study. Furthermore, if the adaptation process proposed here works, we can observe only the adaptive attractor, even if there are potentially multiple attractors (note that the adaptation time is short if the number of attractors is not huge). The experimental/theoretical verification of the existence of multiple attractors in regulatory dynamics remains for future study.

The present noise-induced selection of a higher growth state has not been directly confirmed in real biological systems so far. Standard experimental data concern only the adaptation process based on the signal transduction networks, so that we need novel experimental setups to verify the proposed adaptation mechanism. There are two possibilities. One is the use of artificial gene networks, as demonstrated in [9]. In this approach, one can introduce a gene network disconnected from the existing signal transduction networks, and investigate whether the artificial gene network exhibits selection of a higher growth state. Another possibility is the study of cellular response against environmental changes that the cells have never faced, or response of cells in which known regulatory mechanisms are destroyed. In both cases, by investigating the response of cells, one can examine if cells show adaptive behavior to environmental change, without the sophisticated regulatory mechanisms, but by utilizing the fluctuation-based selection of a higher growth state, as presented in this paper.
